# Detection of titanium particles in human liver and spleen and possible health implications

**DOI:** 10.1186/s12989-018-0251-7

**Published:** 2018-04-11

**Authors:** M. B. Heringa, R. J. B. Peters, R. L. A. W. Bleys, M. K. van der Lee, P. C. Tromp, P. C. E. van Kesteren, J. C. H. van Eijkeren, A. K. Undas, A. G. Oomen, H. Bouwmeester

**Affiliations:** 1 National Institute for Public Health and the Environment (RIVM), Bilthoven, The Netherlands; 2 RIKILT, Wageningen University & Research, Wageningen, The Netherlands; 3 Department of Anatomy, University Medical Center Utrecht, Utrecht, The Netherlands; 4 TNO Earth, Life and Social Sciences, Utrecht, The Netherlands; 5 Present address: Division of Toxicology, Wageningen University, Wageningen, The Netherlands

**Keywords:** Titanium dioxide, Quantification, Human liver, Human spleen, Tissue level, Nanoparticle, Risk assessment, Sp-ICP-HRMS

## Abstract

**Background:**

Titanium dioxide (TiO_2_) is produced at high volumes and applied in many consumer and food products. Recent toxicokinetic modelling indicated the potential of TiO_2_ to accumulate in human liver and spleen upon daily oral exposure, which is not routinely investigated in chronic animal studies. A health risk from nanosized TiO_2_ particle consumption could not be excluded then.

**Results:**

Here we show the first quantification of both total titanium (Ti) and TiO_2_ particles in 15 post-mortem human livers and spleens. These low-level analyses were enabled by the use of fully validated (single particle) inductively coupled plasma high resolution mass spectrometry ((sp)ICP-HRMS) detection methods for total Ti and TiO_2_ particles. The presence of TiO_2_ in the particles in tissues was confirmed by Scanning Electron Microscopy with energy dispersive X-ray spectrometry.

**Conclusions:**

These results prove that TiO_2_ particles are present in human liver and spleen, with ≥24% of nanosize (< 100 nm). The levels are below the doses regarded as safe in animals, but half are above the dose that is deemed safe for liver damage in humans when taking into account several commonly applied uncertainty factors. With these new and unique human data, we remain with the conclusion that health risks due to oral exposure to TiO_2_ cannot be excluded.

**Electronic supplementary material:**

The online version of this article (10.1186/s12989-018-0251-7) contains supplementary material, which is available to authorized users.

## Background

Titanium dioxide (TiO_2_) is produced as titanium white at high production volumes, up to 6 million tons per year [1]. It is incorporated in many products, such as in food (additive E171), toothpaste, supplements and medicines, as well as in applications like paints, plastics, and cosmetics [1]. Food grade TiO_2_ contains a fraction of particles in the nanosize range, which is around 10% number-based [2, 3]. No acceptable daily intake (ADI) for oral ingestion of TiO_2_ has been derived in the past due to the absence of observed toxic effects in the available chronic rodent study [4], the generally assumed negligible uptake of TiO_2_ following ingestion [5], and the assumed insolubility and inertness of the material [6, 7]. Recent human volunteer studies, however, show elevated blood Ti levels (and indications of TiO_2_ particles) 6 h after ingestion of food grade TiO_2_ [8], confirming earlier reports of increased blood Ti-levels after ingestion of 160 nm and 380 nm TiO_2_ particles [9]. Upon evaluating food grade TiO_2_, the European Food Safety Agency (EFSA) acknowledged that TiO_2_ is absorbed after oral application, albeit to a low extent, and transported to various organs [10]. Recently, very low oral (0.02 and 0.6%) absorption of TiO_2_ nanoparticles has been shown in rats, with a retention of these particles in mainly the liver and spleen [11, 12]. This calls for (nano)particle biokinetic studies in humans [13].

Toxicokinetic modelling of TiO_2_ levels in human organs, based on animal studies and accounting for accumulation, has recently led to the conclusion that a human health risk from the oral intake of TiO_2_ nanoparticles cannot be excluded [14]. Although most accumulation was seen in spleen, in the final risk assessment, a potential risk was found for the liver. It remained uncertain whether the modelled levels of TiO_2_ nanoparticles for human liver and spleen are accurate, which is best verified by measurements. Although total-Ti has been detected before in human tissues like liver and spleen [15, 16], there currently are no data on the presence of TiO_2_ (nano)particles in human tissues from people without titanium implants [17, 18]. Here, we present the first *quantitative* measurements of particles, both in size and concentration, in post-mortem liver and spleen of 15 human subjects (see Table 1) with a corresponding assessment of the risks that can potentially be associated with the observed total Ti and TiO_2_ particle concentrations in these tissues.

**Table 1:** Overview of human subjects involved in this study

Subject number	Gender (F/M)	Age (years)	Ethnicity	Ti implants
1	F	80	Caucasian	No
2	F	92	Caucasian	No
3	M	64	Caucasian	Yes
4	M	86	Caucasian	No
5	M	87	Caucasian	No
6	M	79	Caucasian	No
7	F	94	Asian	No
8	F	77	Caucasian	No
9	F	86	Caucasian	No
10	M	77	Caucasian	Yes
11	F	104	Caucasian	No
12	F	96	Caucasian	No
13	F	91	Caucasian	No
14	F	94	Caucasian	No
15	M	56	Caucasian	No

## Methods

Firstly, we determined the total-Ti content in human liver and spleen samples using a fully validated procedure that included the acid digestion of the formaldehyde-fixed homogenized human tissues and ICP-HRMS detection (Peters RJB, Undas A, Memelink J, van Bemmel G, Munniks S, Bouwmeester H, et al.: Development and validation of a method for the detection of titanium dioxide particles in human tissue, submitted). Next, a new, independent subsample was prepared to quantitatively determine the presence of TiO_2_ particles in these tissues. For this, highly sensitive and selective spICP-HRMS was used [2, 19–21]. The enzymatic and gentle chemical sample clean-up and detection method for Ti in tissues and organs was recently fully validated (Peters RJB, Undas A, Memelink J, van Bemmel G, Munniks S, Bouwmeester H, et al.: Development and validation of a method for the detection of titanium dioxide particles in human tissue, submitted). The sample preparation is known not to affect the presence and size of particles [2].

### Samples and sample preparation

The inertness of TiO_2_ allowed the use of livers (15) and spleens (15) obtained from bodies that were donated to the Department of Anatomy of the University Medical Centre Utrecht for educational and research purposes (Table 1). All ethical regulations concerning the use of these organs were followed, and approval for this specific scientific use was obtained from the board of University Medical Center Utrecht. The bodies, 6 men and 9 women who died at the age of 56 to 104 years, had been fixed in 4% formaldehyde. From these persons written informed consent was obtained during life that allowed the use of their entire bodies for educational and research purposes. While there is no information about their diets, it is known that all persons involved are of Caucasian ethnicity except one who was of Asian ethnicity. All have lived in the Netherlands their entire life and it is therefore assumed that most followed a Dutch diet [22]. Of the 15 persons involved, 2 received titanium implants during their lifetime. For sample preparation, each organ was cut into small pieces and grinded to a size of 0.5–1 mm diameter. To investigate potential sample contamination, all materials that had been in contact with the organs were collected. The total-Ti concentrations in these materials or released by these materials were determined. The average of the analytical results of those blank materials were calculated and subtracted from the sample results if they were above the limit of detection (LOD).

### Determination of total-Ti content

An analytical sample of 1 g was collected from each grinded and homogenized sample and brought into a perfluoroalkoxy (PFA) microwave digestion tube to which 6 mL of nitric acid (70% HNO_3_) and 2 mL of hydrofluoric acid (40% HF), were added. All subsamples were digested for 55 min in a MARS microwave system (CEM Corporation, Matthews, NC, USA). The temperature program was as follows: at 1600 W power from 20 to 120 °C in 15 min, then to 160 °C in 10 min, and then to 210 °C in 30 min and hold for 1 min. Following digestion and cooling to room temperature, ultra-pure water was added to a total volume of 50 mL. The extracts were shaken manually, diluted 2 times, and analysed with ICP-HRMS. Quantification was based on ionic titanium standards diluted in the same acidic matrix as the samples. Method blanks were determined by performing the complete procedure, however, without the addition of a sample. The total-Ti content in the blanks was below the method LOD.

### Determination of TiO_2_ particles

For the determination of particle-TiO_2_, a digestion procedure is followed to liberate the particles. This digestion procedure consists of two steps. In the first step, the tissue in the formaldehyde-fixed sample is depolymerized, while in the second step, a standard enzymatic digestion is performed. An analytical sample of 200 mg was collected from the grinded subsamples and brought into a 12-mL PE tube. In the first step, 4 mL of the digestion buffer was added and the sample was vigorously vortexed for 30 s. The digestion buffer was prepared by dissolving 300 mg of Tris buffer, 92.5 mg EDTA, 5 mg SDS and 3 g NaCl in 100 mL of Milli-Q water. Next, 4 g of glycine are added to the solution and mixed with a magnetic stirrer until complete dissolution. This solution was diluted with Milli-Q water to a final volume of 250 mL.

The tube was heated for 3 h. at 100 °C to depolymerize the formaldehyde-fixed tissue. In the second step, and after cooling to room temperature, 910 μL of proteinase K (2.5 mg/ml) was added. The tube was incubated for 16 h. at 37 °C in a shaking water bath. After cooling to room temperature, the digest was diluted with ultra-pure water and analyzed using spICP-HRMS.

### Instrumental analysis with ICP-HRMS

A Thermo Finnigan Element 2 (Thermo Fisher Scientific GmbH, Bremen, Germany), a sector-field based high resolution ICP-MS, was used to measure total-Ti in acidic extracts in standard mode and TiO_2_ particles in single-particle mode (also called time resolved analysis mode). Single-particle ICP-HRMS is a method for the detection and characterization of (nano-)particles [19, 21]. The Thermo Finnigan Element 2 was operated at a forward power of 1300 W and the argon gas flows were at the following settings; plasma, 15.4 L/min; nebulizer, 1.063 L/min; auxiliary, 1.2 L/min. The sample flow rate to the nebulizer was set at 0.5 mL/min. Data acquisition was done in standard mode and in time resolved analysis mode with titanium measured at *m/z* 46.95 in medium resolution mode to avoid interferences from ^36^Ar^12^C, ^32^S^16^O and ^48^Ca. In standard and time resolved mode the dwell time was 250 and 2 ms respectively, with a total acquisition time of 60 s. The transport efficiency was determined by the analyses of a 50 ng/L diluted aqueous RM8013 (60 nm gold nanoparticle) suspension under the same instrumental conditions as the samples but monitoring *m/z* 197 for gold. Finally, single-particle data were exported as csv file and processed in a dedicated spreadsheet for the calculation of particle sizes, particle size distributions, and particle number and mass concentrations. Details about this spreadsheet and the calculation of the parameters can be found elsewhere [19]. Method blanks were determined by performing the complete procedure, however, without the addition of a sample. The mass-based TiO_2_ particle concentrations in the blanks were below the method LOD. Since the blanks of the sampling materials were below the LOD of the total-Ti method they were not involved in the particle analysis.

### LOD

For the total-Ti determination the LOD is calculated as 3 times the standard deviation in the results of a blank sample or a sample with a total-Ti content close to the expected LOD. This sample is analysed on each of the validation days. The LOD is calculated as follows:

$$ LOD=3\times \sqrt{\frac{\sum_{i=1}^k{\left({y}_i-m\right)}^2}{k-1}} $$

where k is the number of samples, y_i_ is the result of a single sample and m is average result of the single samples.

For particle-TiO_2_ there are two LOD values, one for the number- and mass-based concentration (LOD_C_), and one for particle size (LOD_S_). LOD_C_ equals the minimum number of particle peaks in the time scan that differentiates a sample from a blank. A way to determine LOD_C_ is by the IUPAC recommended approximation (Poisson) described as [23],

$$ {LOD}_C=3.29\times \sqrt{N}+2.72 $$

where N is the number of particle peaks observed in the time scan of a blank. The particle number LOD_C_ can be converted into mass units if the size and density of the particle are known. The determination of the LOD_S_ is described by Lee et al. and can be estimated as follows [24],

$$ {LOD}_S=\sqrt[3]{\frac{6\times 3{\sigma}_m}{R\times {f}_a\times \rho \times \pi }} $$

where σ_m_ is the standard deviation in the background noise in the time scan, R is the ICP-MS response (cps/μg), f_a_ is the mass fraction of analysed element in the nanoparticle and ρ is the density of the nanoparticle material (g/cm^3^). The upper size limit of detection is estimated to be around 1500 nm.

### Confirmation of TiO_2_ particles wit SEM-EDX

Two subsamples of the grinded sample material of both the livers and the spleens were studied using scanning electron microscopy with energy dispersive X-ray detection (SEM-EDX) to confirm the presence of TiO_2_ particles in human liver and spleen. The samples with the highest TiO_2_ concentrations (as determined with ICP-HRMS) were selected for confirmation with SEM-EDX. Typically, subsamples of > 100 tissue grains were collected on a sampling stub and dried to remove water. These subsamples were analysed with a high resolution field emission gun scanning electron microscopy in combination with energy dispersive X-ray analysis (FEG-SEM/EDX). Approximately 500 images for each sample were viewed at different magnifications (5.000–100.000 X) to identify TiO_2_ particles. For each sample, approximately 10 TiO_2_ particles (single nanoparticles as well as aggregates/agglomerates) were detected. The surface of the grains was systematically analysed for TiO_2_ particles using the backscattered electron imaging mode. After detection of particles in a field of view, X-ray spectra from the detected particle and surrounding matrix were acquired to determine the identity. Subsequently, plasma-ashing was applied to remove the lipid fraction and obtain a sharper image of the TiO_2_ particle.

## Results

All tissue levels are given as wet organ weights as obtained after fixation in formaldehyde.

### Total Ti measurements

As shown in Table 2, the total-Ti content in the liver ranged from 0.02 to 0.09 mg Ti/kg tissue with an average value of 0.04 ± 0.02 mg Ti/kg tissue^1^. For spleen, the total-Ti content ranged from 0.02 to 0.4 mg Ti/kg tissue with an average value of 0.08 ± 0.1 mg Ti/kg tissue^1^. In the sparsely available literature on human data, liver and spleen concentrations ranging between 0.2 and 1.9 mg Ti/kg tissue have been detected. These concentrations have been measured using X-ray fluorescence and neutron activation analysis, while we used HR-ICPS [25, 26].

**Table 2:** Ti and TiO_2_ particles in human (post mortem 4% formaldehyde fixed) liver and spleen

		Total Ti	TiO_2_ (Particles)		Ti in particles^a^		Total Ti	TiO_2_ (Particles)		Ti in particles^a^
Human	Tissue		size range	number of particles	min - max	Tissue		size range	number of particles	min - max
subject		mg/kg	nm	10^9^ /kg tissue	mg/kg tissue		mg/kg	nm	10^9^ /kg tissue	mg/kg tissue
1	Liver	0.04	85–320	2.3–7.2	0.01–0.04	Spleen	0.1	90–580	5.7–18	0.06–0.2
2	Liver	0.09	90–440	6.6–21	0.08–0.3	Spleen	0.4	90–420	18–56	0.1–0.4
3	Liver	< LODt	< LODs	< LODn	< LODc	Spleen	0.02	85–370	1.2–3.8	0.01–0.04
4	Liver	0.05	85–550	1.4–4.4	0.03–0.1	Spleen	0.09	85–320	2.8–8.8	0.01–0.02
5	Liver	< LODt	< LODs	< LODn	< LODc	Spleen	0.03	85–520	1.5–4.7	0.02–0.07
6	Liver	0.03	85–380	2.1–6.6	0.01–0.04	Spleen	0.02	85–350	1.3–4.1	0.01–0.04
7	Liver	< LODt	85–370	1.3–4.1	0.01–0.02	Spleen	0.02	< LODs	<LODn	< LODc
8	Liver	0.02	< LODs	< LODn	< LODc	Spleen	< LODt	< LODs	<LODn	< LODc
9	Liver	< LODt	< LODs	< LODn	< LODc	Spleen	0.2	85–410	9.3–29	0.08–0.3
10	Liver	< LODt	< LODs	< LODn	< LODc	Spleen	0.02	85–360	2.1–6.6	0.01–0.04
11	Liver	0.04	85–450	2.6–8.1	0.02–0.07	Spleen	0.03	90–420	3.2–10	0.02–0.07
12	Liver	0.02	< LODs	< LODn	< LODc	Spleen	0.04	90–720	2.1–6.6	0.05–0.2
13	Liver	< LODt	90–440	1.0–3.1	0.03–0.1	Spleen	0.02	90–390	2.3–7.2	0.03–0.10
14	Liver	< LODt	< LODs	< LODn	< LOD	Spleen	0.03	90–430	2.7–5.3	0.03–0.1
15	Liver	< LODt	< LODs	< LODn	< LODc	Spleen	0.04	90–500	2.4–7.5	0.03–0.1
	n > lod	7	7–7	7–7	7–7		14	13–13	13–13	13–13
	average	0.04^b^	86–421	2–8	0.03–0.1		0.08 ^c^	88–445	4–13	0.04–0.1
	mode	0.04	85–440	–	0.01–0.04		0.02	85–420	2–7	0.01–0.04
	stdev	0.02	2–74	2–6	0.02–0.1		0.1	3–110	5–15	0.03–0.11
	min	0.02	85–320	1–3	0.01–0.02		0.02	85–320	1–4	0.01–0.02
	max	0.09	90–550	7–21	0.08–0.3		0.4	90–720	18–56	0.1–0.4

Particle TiO_2_ concentrations are reported as measured (min) and after correction for the analytical recovery (max). All concentrations are corrected for total concentrations in blanks (0.05 mg/kg). LODt (total-Ti) = 0.01 mg/kg; LODs (size) = 85 nm; LODn (number) = 0.8 × 10^9^/kg; LODc (calculated Ti in particles) = 0.005 mg/kg; ^a^calculated Ti in particle, calculated according to Laborda et al. [21] and Peters et al. ^b^ if calculated with ½ LOD for samples below LOD, average total-Ti = 0.02 mg/kg;^c^if calculated with ½ LOD for samples below LOD, average total-Ti = 0.07 mg/kg

The blank-corrected limit of detection (LOD)_total Ti_ was 0.01 mg/kg tissue, while the analytical recovery for total-Ti was 112 ± 34%, which is in the range of accepted analytical standards [20]. None of the specific steps in the tissue and sample preparation contributed to the blank total-Ti. Two human subjects carried a titanium implant, the total-Ti content in liver and spleen of these subjects was comparable to those observed in the liver and spleen in other subjects.

### Particle measurements

**Fig. 1:**
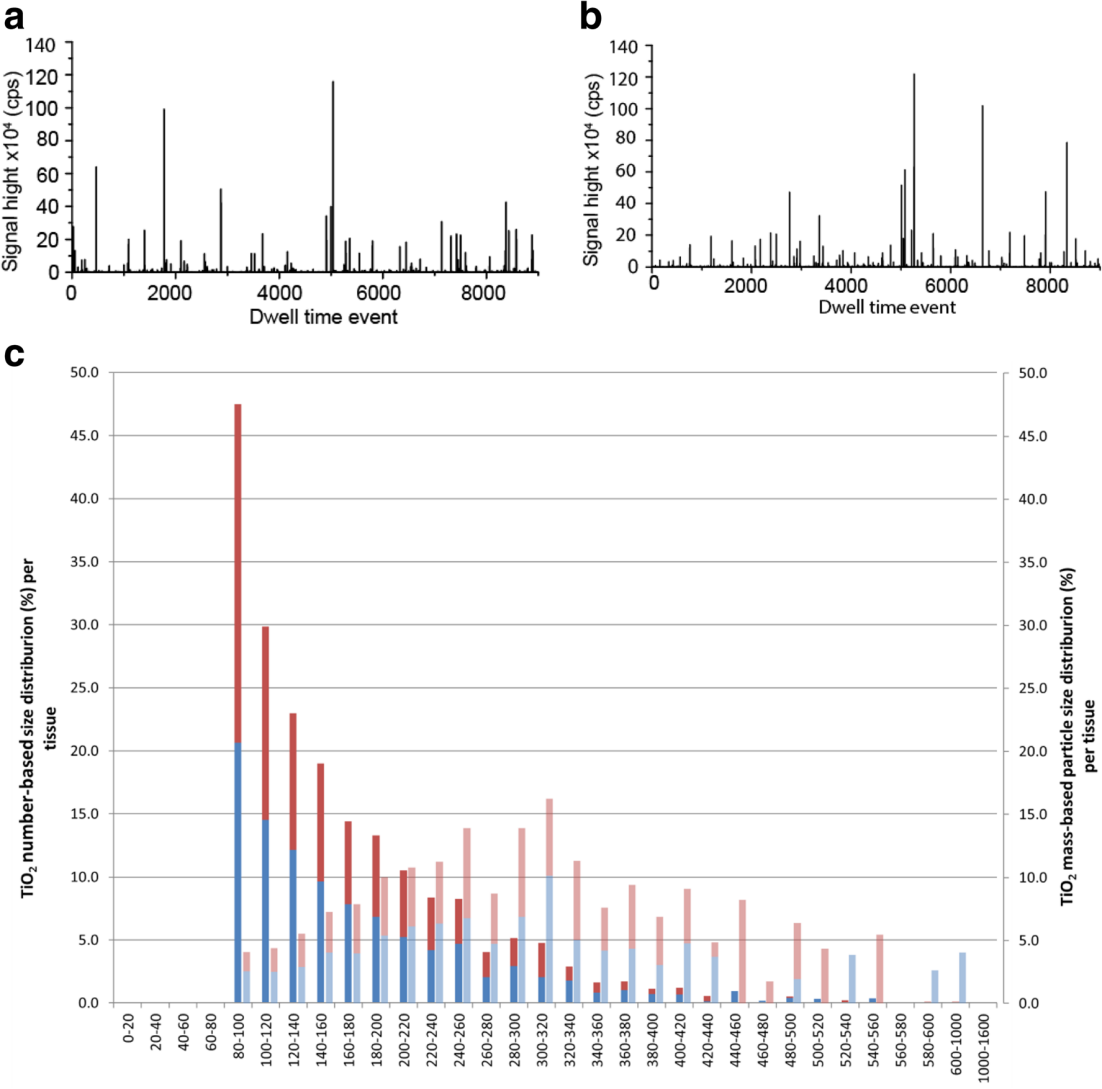
TiO_2_ particles in human (post mortem) liver and spleen. Time scans of the spICP-HRMS analyses of a liver sample (**a**) and spleen sample (**b**). The number of spikes in the time scan is directly proportional to the number of particles in the sample. The signal height of the peaks is directly proportional to the particle’s mass from which the equivalent spherical particle size is calculated [22, 23]. **c** The calculated number-based particle size distribution (left axis and dark colours) and the calculated mass-based particle size distribution (right axis and light colours). Since the particle size distribution in liver (red bars) and spleen (blue bars) are very similar, they are stacked

The presence of TiO_2_ particles in the tissues is evidenced by the characteristic spikes in the time scans of the spICP-HRMS analysis of liver and spleen samples (Fig. 1a, b). TiO_2_ particles were detected in 7/15 liver and 13/15 spleen samples (Table 2). The smallest TiO_2_ particle that can be detected with this method (LOD_size_) in these tissues is 85 nm. The number-based TiO_2_ particle size distributions in liver and spleen were comparable and had a size range of 85–550 and 85–720, respectively (Table 2 and Fig. 1c). SpICP-HRMS does not allow a further characterisation of the particles being present as agglomerates, aggregates or primary particles. In the tissues, 24% of the TiO_2_ particles in the number-based size distribution was < 100 nm, but this fraction may be underestimated considering the LOD_size_ of 85 nm.

The TiO_2_ particle mass concentration in liver ranged from 0.01 to 0.3 mg Ti/kg tissue (1.0 × 10^9^ to 21 × 10^9^ TiO_2_ particles/kg tissue) (Table 2). In spleen, this concentration ranged from 0.01 to 0.4 mg Ti/kg tissue (1.2 × 10^9^ to 56 × 10^9^ TiO_2_ particles/kg tissue). The LOD_particle-number_ in the tissue matrix is 0.8 × 10^9^ particles per kg tissue. The analytical recovery of TiO_2_ particles by enzymatic digestion of the matrix is 32 ± 7%. This low analytical recovery is in accordance with best international practices for this sample preparation and detection technique [27]. Because of the low analytical recovery, the Ti concentration of the particles is presented both as a minimum (not corrected for analytical recovery), and a maximum (corrected for analytical recovery) (see Table 2).

The total-Ti values are in general within this Ti concentration range in the particles, Furthermore, tissues with high total-Ti concentrations also contained high TiO_2_ particle concentrations, and vice versa. Based on the maximum Ti concentration values in particles, on average minimally 51% (liver) and 67% (spleen) of total Ti is present in these tissues as particle. Taking into account the analytical recovery (32 ± 7%) and the LOD_size_ (85 nm) for the particles measurements, we assume all total Ti is present as particles.

We did not observe a correlation in the abundance of the TiO_2_ particles in liver and spleen from the same subjects, while this would be expected based on the shared external exposure. The reason for this lack of correlation may be related to inter-individual differences in the various involved biodistribution processes.

**Fig. 2:**
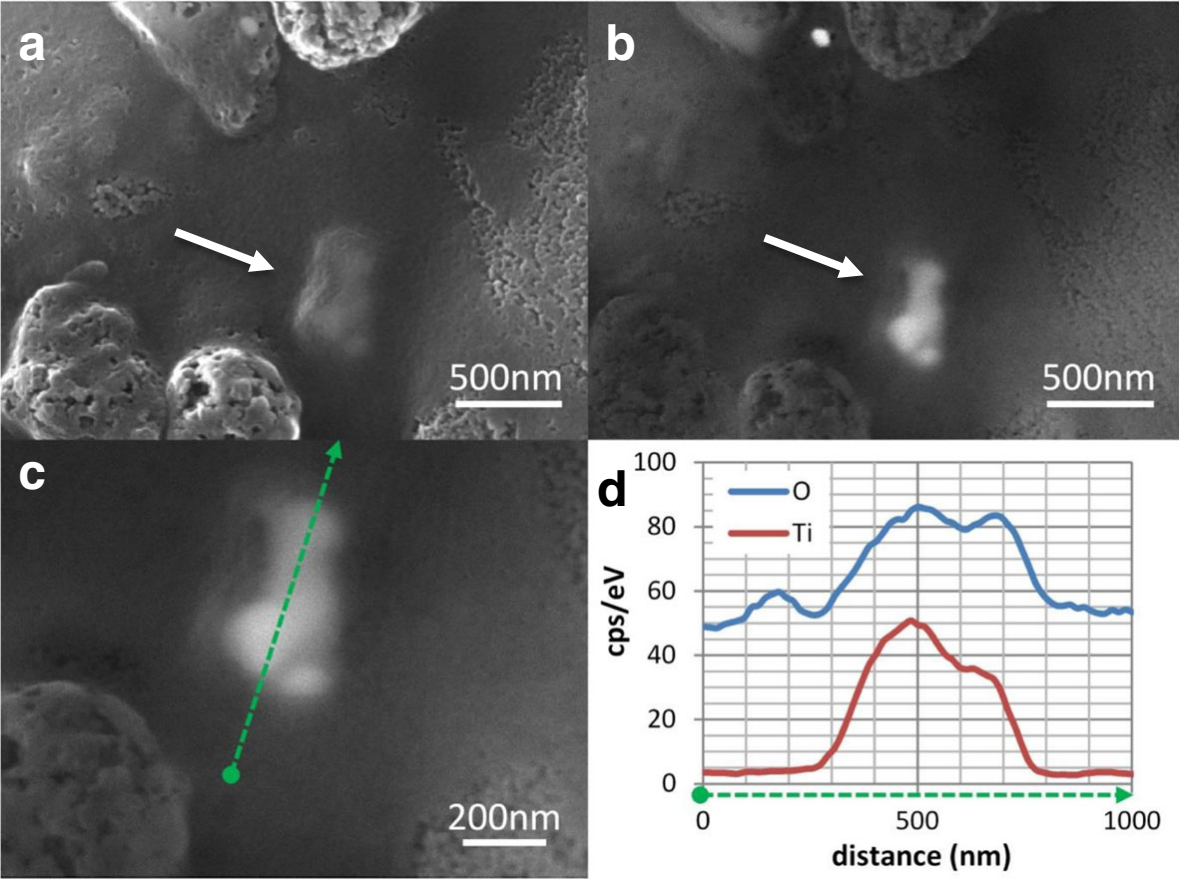
SEM characterization of detected TiO_2_ particles in a dried liver sample. **a** The secondary electron microscope image shows a TiO_2_ agglomerate below the surface of the liver tissue (arrow). **b** The backscattered electron image reveals the spherically shaped primary particles within the agglomerate (arrow), with diameters between 75 and 150 nm; (**c**) illustrates the path of the EDX line scan across the aggregate in the same image at higher magnification; (**d**) demonstrates the presence of TiO_2_ based on the corresponding increase of response for Ti (red line) and oxygen (blue line) at the position of the particle. This forms a clear indication that the detected particle is indeed TiO_2_

Lastly, small tissue grains of liver and spleen from two subjects were analysed using SEM-EDX to visualize the TiO_2_ particles. As shown in Fig. 2, the observed particles are composed of Ti and oxygen and are present as an aggregate or agglomerate, consisting of smaller primary particles of 75–150 nm. Presence of Ti was also confirmed semi-quantitatively by EDX analysis in dry-ashed liver and spleen samples (Fig. 2d).

Together, these analyses show that approximately all TiO_2_ is present as particles in human liver and spleen, with sizes ranging 85–550 and 85–720, respectively (upper size limit of detection was > 1.5 μm). Probably also smaller particles are present, however these cannot be detected with the current methods. The SEM analysis of the particles suggests that the larger particles consist of smaller primary particles. Therefore, for the purpose of risk assessment, we assume that all Ti is ultimately present as TiO_2_ nanoparticles.

### Risk assessment

**Fig. 3:**
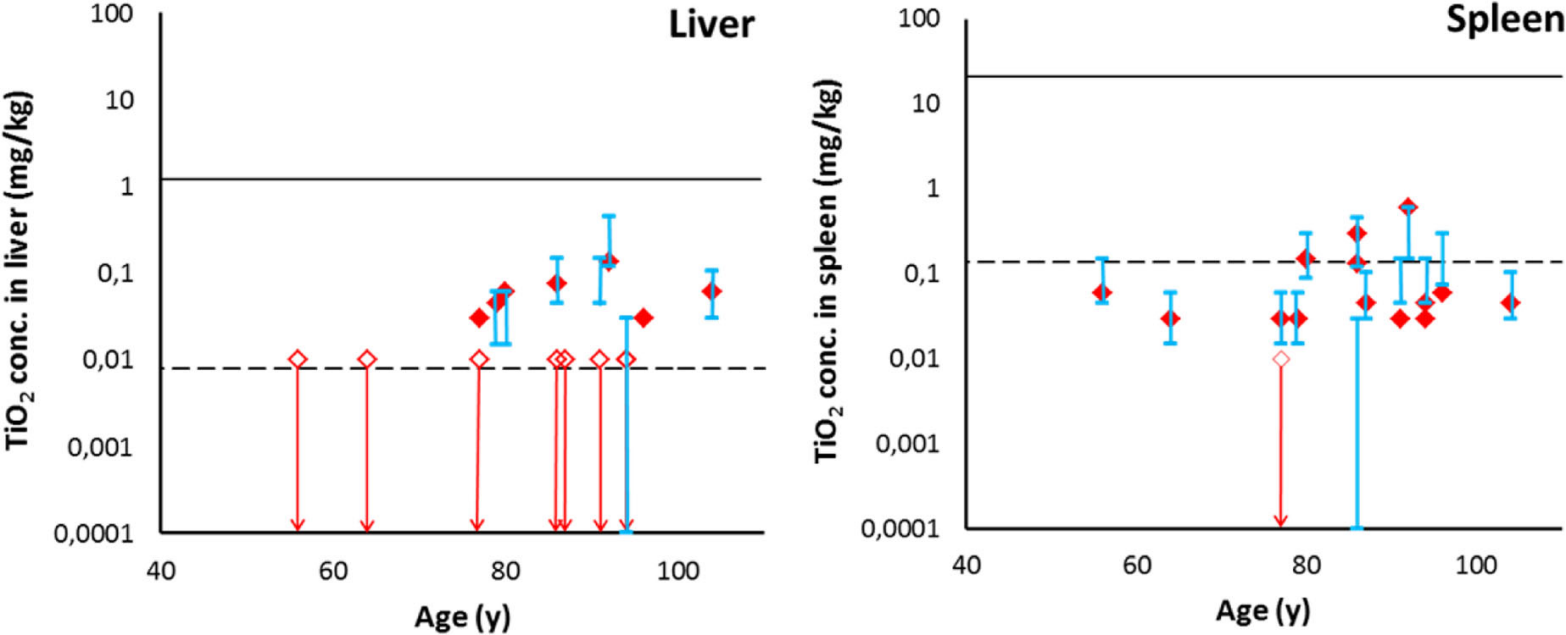
Observed liver and spleen concentrations compared to toxicological effect levels. Measured total Ti (expressed as TiO_2_ to enable comparison, red diamonds) and TiO_2_ particle concentrations (blue ranges) in human liver and spleen are plotted against age, together with liver or spleen concentrations that are relevant for risk assessment (black lines). Open diamonds and arrows represent the possible levels in the samples where the Ti level was below the limit of detection (LOD). Measured particle concentrations are given as a range between the minimum possible level (no correction for the analytical recovery) and the maximum possible level (corrected for the analytical recovery). The solid black line represents the organ level at the No Observed Adverse Effect Level (NOAEL) in the animal study, i.e. the highest level at which no adverse effect was observed. The dashed black line represents the organ level below which no effects are expected in humans, considering several uncertainties in the animal data

In a next step, the total TiO_2_ levels in liver and spleen were compared to the toxicologically safe tissue levels for TiO_2_ (0.14 mg/kg for spleen and 0.008 mg/kg for liver), as reported earlier [14]. For liver, the measured TiO_2_ concentrations are all below the level where effects occurred in animals, which were the occurrence of liver edema and liver enzyme level changes. However, the seven measurements >LOD are above the level at which effects may occur in humans (Fig. 3). For the estimation of the safe level in humans, interspecies differences were considered and sensitive subpopulations were accounted for, which would include children, elderly, and diseased people (see also Additional file 1). It can therefore not be excluded that the observed liver levels lead to adverse effects in humans, such as a liver functioning less well, leading to e.g. less detoxification of substances in the blood, and less albumin production. For spleen, it is unlikely that adverse effects will occur in humans as the measured levels are distributed around the estimated safe levels (please note that in the key toxicological study [28], no adverse effects were reported, thus the highest tested dose was used here [14] (see also Additional file 2).

## Discussion

The TiO_2_ particles observed in the human liver and spleens may have entered the body through dermal, inhalatory or oral exposure. No data on exposure, and thus neither on exposure route, of the subjects included in this study during life is available. However, dermal uptake of TiO_2_ particles is unlikely, as TiO_2_ particles do not penetrate the (intact) human skin [17, 29]. It is likely that inhalatory uptake can be neglected as the chance is small that these people (all) had occupations with TiO_2_ exposure through air. In addition, the estimated maximal non-occupational exposure through this route is 4.5 μg Ti/day (with an average of 0.75 μg TiO_2_/day; based on the Ti concentrations in non-occupational settings of 0.01–0.1 μg/m^3^) [30]. Furthermore, most of these inhaled TiO_2_ particles are eliminated from the lungs by mucociliary clearance in the ciliated part of the lungs, and subsequently swallowed, as seen in some studies [31, 32]. Probably, most human subjects followed a West European diet and used toothpaste, which may result in a mean oral intake of 0.06–5.5 mg TiO_2_/kg body weight/day [3, 10, 33, 34]. Recent human volunteer studies indicate the systemic uptake following ingestion of TiO_2_ particles [8, 9]. Strikingly, the size range of the TiO_2_ particles in the human livers and spleens (i.e. 86–421 and 88–445 nm, respectively) falls within that of the TiO_2_ particles in food products (30–600 nm diameter [2]). In conclusion, intestinal exposure, e.g. from food, toothpaste and supplements, but also from any inhaled and swallowed particles, is the most likely source of the Ti and TiO_2_ particles as found in the liver and spleens of these 15 subjects. This justifies our comparison with safe tissue levels derived from oral toxicity studies.

The current study shows that both the element Ti and TiO_2_ particles are present in post mortem fixed human liver and spleen and that health risks from liver damage due to oral exposure to TiO_2_ still cannot be excluded, especially in elderly people. Clearly, some issues as addressed in Heringa et al. [14] remain unresolved, like the limitations in the toxicological data set and the impact that different forms of TiO_2_, with different size [35], surface properties or crystalline structure can have on the observed toxicity. In addition, the available organs for this study were, understandably, limited to relatively older people and their TiO_2_ exposure and health condition is not known. Recently, concern has been raised on the potential contribution of TiO_2_ on tumor formation in the intestine [36, 37]. More information on the adverse effects of TiO_2_ particles, including potential effects on liver as well as on potential carcinogenic induction and promotion in the gastrointestinal tract, would reduce the uncertainties in the current risk assessment.

## Conclusion

Using two independent particle characterization techniques, we unequivocally show the presence of TiO_2_ particles in (post mortem) human liver and spleen and provide quantitative data on the total human organ burden of TiO_2_ particles for the first time. Particles with a size between 85 and 720 nm were found in tissue, of which at least 24% was smaller than 100 nm. This unique study thereby adds another critical piece to the risk assessment puzzle for TiO_2_ (nano)particles, showing that health risks related to liver damage (i.e. liver edema and liver enzyme changes) due to TiO_2_ particles still cannot be excluded.

## Additional file

Additional file 1: Supplementary Information for "Detection of titanium particles in human liver and spleen and possible helath implications". (DOCX 130 kb)

## Abbreviations

(FEG-)SEM-EDX: (field emission gun) scanning electron microscopy – energy dispersive X-ray
(sp)ICP-HRMS: (single particle) inductively coupled plasma high resolution mass spectrometry
ADI: Acceptable daily intake
EDTA: Ethylenediaminetetraacetic acid
EFSA: European Food Safety Authority
HF: Hydrofluoric acid
HNO_3_: Nitric acid
IUPAC: International Union of Pure and Applied Chemistry
LOD: Limit of detection
NaCl: Sodium chloride
NOAEL: No observed adverse effect level
PE: Polyethylene
PFA: Perfluoroalkoxy
SDS: Sodium dodecyl sulphate
Ti: Titanium
TiO_2_: Titanium dioxide

## Footnotes

1 Values reported here are based on values above LOD, excluding the values below LOD.
