# Supplementary material for: Detection of titanium particles in human liver and spleen and possible health implications
Source: Part Fibre Toxicol. 2018 Apr 11;15:15. doi: 10.1186/s12989-018-0251-7 (PMC5896156; doi:10.1186/s12989-018-0251-7)
Supplement: Supplementary file 1 — Supplementary Information for "Detection of titanium particles in human liver and spleen and possible helath implications". (DOCX 130 kb) [file 12989_2018_251_MOESM1_ESM.pdf]

Supplementary information for:

Detection of titanium particles in human liver and spleen and possible health implications

M.B. Heringa[1], R.J.B. Peters[2], R.L.A.W. Bleys[3], M.K. van der Lee[2], P.C. Tromp[4], P.C.E. van Kesteren[1], J.C.H. van Eijkeren[1], A.K. Undas[2], A.G. Oomen[1], H. Bouwmeester[2]

[1] National Institute for Public Health and the Environment (RIVM), Bilthoven, The Netherlands

[2] RIKILT – Wageningen University & Research Centre, Wageningen, The Netherlands

[3] Department of Anatomy, University Medical Center Utrecht, Utrecht, The Netherlands

[4] TNO Earth, Life and Social Sciences, Utrecht, The Netherlands

# current address: Division of Toxicology, Wageningen University, The Netherlands.

1. Supplemental analytical results

Table S1 shows the percentage of Ti mass detected as particle, when comparing the results from the amount of Ti present as particle versus the amount of total-Ti as present in the same tissue material (note, two independent methods). Tissues with high total-Ti concentrations also contained high TiO_2 particle concentrations, and *vice versa*. On average 51% (liver) and 67% (spleen) of total Ti is present in the tissue as particle when based on the data not corrected for the analytical recovery. When the recovery for the particle detection is taken into account (32 ± 7%), on average 188% (liver) and 240% (spleen) of the total Ti in tissue is present as particle. These high values (>100%) reflect an extreme in the measurement uncertainties, where the true value is probably somewhere in between the found percentages, i.e. around 100%. Therefore, taking into account the analytical recovery, measurement uncertainties and the LOD_{size} (80nm) for the particles measurements, we assume all total Ti is present as particles.

Table S1. Percentage of Ti mass detected as particle.
Particle TiO_2 concentrations are reported as measured (min) and after correction for the analytical recovery (max). See Table 2 in main document for the amount of Ti present as particle and the amount of total-Ti in each tissue.

	% Mass Ti present as particle	
**Number**	**Liver**	**Spleen**
1	25 - 100	60 - 200
2	89 - 333	25 - 100
3	<LOD	50 - 200
4	60 - 200	11 - 22
5	<LOD	67 - 233
6	33 - 133	50 - 200
7	<LOD	<LOD
8	<LOD	<LOD
9	<LOD	40 - 150
10	<LOD	50 - 200
11	50 - 175	67 - 233
12	<LOD	125 - 500
13	<LOD	150 - 500
14	<LOD	100 - 333
15	<LOD	75 - 250
Average	51-188	67 - 240
Modus		50 - 200
St dev	25-90	39 - 137
min	25-100	11 - 22
max	89-333	150 - 500

2. Toxicity data

Since the submission of Heringa et al.[1], new studies have been published, which have been evaluated for their impact on the current paper.

Bettini et al. (2017)[2] reported an effect in the spleen of rats exposed to NM-105 or E171 for 7 days at 10 mg/kg bw/d (see Table S3): increased production (compared to controls) of IFN-γ and IL-17 when spleen cells were isolated from the rats and cultured with anti-CD3/CD28 antibodies to induce cytokine secretion. This contrasts with the lack of observed effects in the 30-d study of Wang et al.[3] at up to 200 mg/kg bw/d of 75 nm anatase TiO_2. Please note that Wang et al.[3]did not determine IFN-γ and IL-17 levels. This isolated biochemical effect at one single dose after only 7 days suggest that immunotoxic effect may occur, but would need to be reproduced and further substantiated in other studies before it can outweigh the result of Wang et al.[3] and be used in risk assessment. If 10 mg/kg bw/d would be used as the LOAEL for the spleen, the outcome would again be the same as currently found for the liver effects: the spleen levels would fall between the level where the effect was found in rats and the level

regarded as safe for humans. The conclusion would therefore still be that a health risk for TiO_2 cannot be excluded. We do recommend further study of these observed spleen effects.

The same study[2], as well as Urrutia-Ortega et al.[4] found an increase in preneoplastic lesions in the intestines of rats and mice, respectively, exposed orally to a 5 mg E 171/kg bw/d. While these indications warrant further research, they are outside of the scope of this paper, which focusses on spleen and liver. These effects have therefore not been further considered here.

Table S2. Details of key study and supportive study selected earlier and a new study, with toxicological data on liver and spleen for TiO_2 nanoparticles (NPs).For an overview of more toxicity studies and further details on the assessment of the key toxicity studies, see Heringa et al. [1].

**Study details**	**TiO_2 particle details[1]**	**External dose at onset of adverse effects[2]**	**Critical effects at LOAEL (not exhaustive)[3]**	**Source**
• 30 days<br>• Sprague Dawley rats, males only<br>• 7 animals/dose<br>• 0; 10; 50; 200 mg/kg bw/day<br>• Intragastric in water<br>• Young rats (3 weeks old) and adult rats (8 weeks old)	• Anatase<br>• 75 ± 15 nm average diameter<br>• SSA: 63.95 m^2/g (BET)	*Liver young:*<br>*NOAEL = 10 mg/kg bw/d*<br><br>*Liver adult:*<br>*NOAEL = 50 mg/kg bw/d*<br><br>*Spleen (young + adult):*<br>*NOAEL = 200 mg/kg bw/d*	Young rats:<br>Liver edema;<br>AST (-27%) and ALT/AST (+61%; ALT unchanged) → liver damage<br><br>Adult rats:<br>TBIL (-24%) → liver damage (multiple possible causes)	3<br>(key study)
• 14 days<br>• Swiss albino mice, males only<br>• 5 animal/dose<br>• 0; 10; 50; 100 mg/kg bw/d<br>• Oral in Milli-Q water	• Anatase<br>• 2-50 nm primary size<br>• Hydrodynamic diameter varied per dose: 281, 294 and 301 nm, respectively<br>• SSA not given	*LOAEL = 10 mg/kg bw/d*	ALT (+13%) and ALP (+78%)→ liver damage<br>Olive tail moment (comet assay) ↑ → genotoxicity	5<br>(supportive study)
• 7 days<br>• Wistar rats, adult males only<br>• 10 animals/dose<br>• 0; 10 mg/kg bw/day<br>• Intragastric in water	NM-105<br>• Anatase:Rutile (84:16)<br>• Anatase 24.3±3.5 nm / Rutile 15.0 nm (primary particle size, by TEM)<br>• SSA: 46 m^2/g (BET)<br><br>E171From French commercial supplier of food colourants. No further information.	*Spleen: "LOAEL" = 10 mg/kg bw/d*	For both NM-105 and E171: increased production (compared to controls) of IFN-γ and IL-17 when spleen cells were isolated from the rats and cultured with anti-CD3/CD28 antibodies to induce cytokine secretion → more potent Th1/Th17 immune response than in controls.	2<br>(new study)

[1]SSA = specific surface area; BET = Brunauer-Emmett-Teller adsorption method
[2] the NOAEL (No Observed Adverse Effect Level) is the highest experimental dose at which no adverse effects start to occur , the LOAEL (Lowest Observed Adverse Effect Level) is the lowest experimental dose at which adverse effects have been observed in the animals. As Point of Departure (PoD) for determining what is an estimated safe dose in humans, the lowest NOAEL is usually selected
[3] AST = aspartate transaminase; ALT = alanine transaminase; TBIL = total bilirubin; ALP = alkaline phosphatase; IFN-γ = interferon-gamma; IL = interleukin; CD = cluster of differentiation; Th = T-helper cell

3. Explanation of toxicological risk assessment procedure

For the assessment of a toxicological human health risk, the actual exposure is compared to the determined safe exposure[6]. This can be either based on the external exposure, such as an ingested amount per day per kilogram bodyweight, or based on an internal exposure, such as a concentration in a tissue. In this study, a comparison based on internal exposure is performed, where the actual internal exposure in liver and spleen of humans has been measured. The safe internal exposure is determined from animal study data, starting preferably with the highest experimental dose at which no adverse effects have been observed (the No Observed Adverse Effect Level (NOAEL)). If this is not available, a LOAEL (Lowest Observed Adverse Effect Level) can be used, which the lowest experimental dose at which adverse effects have been observed in the animal. Using a toxicokinetic model, based on toxicokinetic data of the substance, it can be calculated what the concentration in a certain human tissue is, if a human being would be exposed to this NOAEL (or LOAEL).

This internal NOAEL then further needs to be corrected for interspecies differences, intraspecies differences, exposure duration differences, data quality concerns and other differences and concerns, where applicable. Interspecies differences relate to the differences between animals and humans and intraspecies differences relate to differences between sensitive or susceptible humans (e.g. children, elderly and diseased people) and healthy, unsusceptible humans. The correction for exposure duration is applied when the data come from tests that did not expose the animals for their entire lifetime (i.e. 2 years for rats and mice), but only 28 days, for example. Typical correction factors (also named uncertainty factors or assessment factors) are 10 for interspecies differences between rat and human, 10 for intraspecies differences, 6 for extrapolation from a 28-d test to a 2-y duration and 3 for using a LOAEL instead of a NOAEL. The internal NOAEL is divided by these factors to obtain the internal tissue level that is deemed safe for the human population for their entire lifetime. The precise derivation of the safe internal tissue levels for TiO_2 nanoparticles, including the applied correction factors, is described in Heringa et al.[1].

4. Comparison of measured liver and spleen concentrations with model estimates

Besides a comparison of the measured organ concentrations with safe levels to determine whether there is a health risk, the measured organ concentrations were also compared to the estimated organ concentrations to see if these estimations were relatively accurate. These estimated organ concentrations originate from our previous risk assessment[1]. They are based

on the P95 and P50 of the Dutch intake of TiO_2 NPs from food and toothpaste as estimated by Rompelberg et al.[7] and a toxicokinetic model[1]. The P95 (or P50) means that 95% (or 50%) of the population has an intake equal to or lower than the P95 (or P50) value, though it is indicated in this paper that the intake estimations might be underestimated as medicines were not included, and it was assumed in the intake estimation that people are not brand-loyal.

Figure S1 shows the estimated human liver and spleen levels (dotted grey lines) and measured concentrations (symbols), together with the safe levels (black lines). The trend changes in the estimated levels (e.g. change in slope of the dotted grey curves) seen at ages 7, 18 and 70 are caused by the difference in intake levels between the three age groups for which intakes were assessed (age groups 2-6 years, 7-69 years and >70 years old) and by the growth stop at around age 18. In real life, the change in intake with increasing age and growth will be gradual.

The number (or mass) of TiO_2 particles in post mortem human spleen, in the samples >LOD, scatter around the estimated level corresponding to the P50 of the intake (i.e. "the P50 level"), with one TiO_2 measurement just exceeding the P95 level, and one measurement below the detection limit. The variation between the measured spleen levels is of the same order of magnitude as the variation in the population modelled, e.g. considering the difference using the P50 and P95 intake. In the human liver, the measured levels of TiO_2 particles (for the seven samples above the LOD) are 10-100-fold higher than the predicted P50 levels and up to a factor 10 higher than the predicted P95 levels. In half of the liver samples, the TiO_2 levels were below the detection limit, therefore, these could be closer to the P50 values predicted by the model than the other samples. Due to the number of samples (8 out of 15) in which no TiO_2 could be quantified, only a limited comparison of the observed and modelled variation could be made. This limited comparison seems to indicate a similar order of magnitude in the variation in the measured samples as in the population modelled.

The measured data show no increase of the organ levels with age, while this would be expected in the spleen, as the toxicokinetic data indicated accumulation in the spleen without any elimination. In the liver, some elimination was seen in the toxicokinetic data, leading to a predicted steady state in the TiO_2 level in liver after the age of ~20 years (see figure S1). Due to the relatively high age of the organ donors, steady state would have been reached and no differences in TiO_2 levels would be expected between the different donors. The fact that no accumulation is clear in the spleen either, can be explained by comparing the observed

variation in the measured data to the expected increase in the estimated levels due to the accumulation. After the first few years of life, the expected increase due to accumulation is smaller than the variation, thus accumulation will not be visible due to the variation in this set of data from 15 spleens from individuals of high age only.

Considering the data limitations and that some important pieces of information are still unknown, the measured data show our previous estimations were quite good. Complete accuracy cannot be expected with the currently available information. Examples of data gaps and limitations are: the unknown actual intake of the organ donors, the limited number of data points and the absence of information for individuals younger than 58 years. In addition, it was ignored that TiO_2 intake may have been affected in the years before the organ donor's death due to limited food intake due to serious illness or increased through medicinal pills. For the continuous accumulation in the spleen (i.e. no steady state expected there), it may have mattered that the donors of the analyzed organs were mostly born before the 60's, when TiO_2 was allowed to be used as food colourant.

Apart from these general model limitations and limited knowledge on donor exposure, the observed underestimation for liver levels by the model might possibly have been caused by the data underlying the toxicokinetic model as these were intravenous (IV) data from rat, not oral data from humans. After oral exposure of chemicals, there is a first pass of the liver after uptake in the blood, which could lead to a relatively higher absorption in the liver at oral exposure than after intravenous dosage. It is doubtful, however, that nanoparticles such as those of TiO_2 follow the same route as dissolved chemicals, as they seem to be absorbed in the gut primarily through the M-cells in Peyer's patches[8,9]), which pass the particles to the lymphatic system instead of the blood. The recent oral and IV toxicokinetic data of Kreyling et al.[10,11] suggest there may be differences in the distribution of TiO_2 NPs between administration routes. However, these studies do not show relatively higher liver levels after oral administration than after IV administration, rather the opposite, and thus cannot explain that we find higher liver TiO_2 levels than estimated. Other considerations to explain this difference with our estimations are interspecies differences and the fact that the toxicokinetic data for only four different TiO_2 forms were available and included in the toxicokinetic model[1]. Limited information is available on the exact properties of the TiO_2 consumed in daily life (size, crystallinity, surface properties), and on whether the toxicokinetic model can predict the toxicokinetic behavior of precisely these forms of consumed TiO_2 particles. This may therefore also cause differences between the modelled and measured liver concentration.

The modelled levels were based on an assumed absorption of 0.02%[12], after an analysis of all available information at the time[1]. Recently, Kreyling et al. have determined an oral absorption of 0.6% for 70 nm TiO_2 particles using radiolabeling, which is 30-fold higher[11]. If this would be the true absorption percentage in humans and used in the modelling, the measured liver levels would correspond to the predicted range of liver levels by the kinetic model and estimated Dutch intake. However, then the measured spleen levels would fall 10-100-fold below the predicted range, which could be explained by a higher elimination from the spleen than currently known.

Clearly, the different values found in the currently available toxicokinetic studies in animals and the absence of human biokinetic data of TiO_2 provide uncertainty in the prediction of human organ levels, as also discussed by Bello &Warheit[13]. This also means that the currently presented measured levels in human organs are of high value for the risk assessment of TiO_2 NPs. The calculated intake and toxicokinetic model can be used indicatively for further risk assessment work on TiO_2.

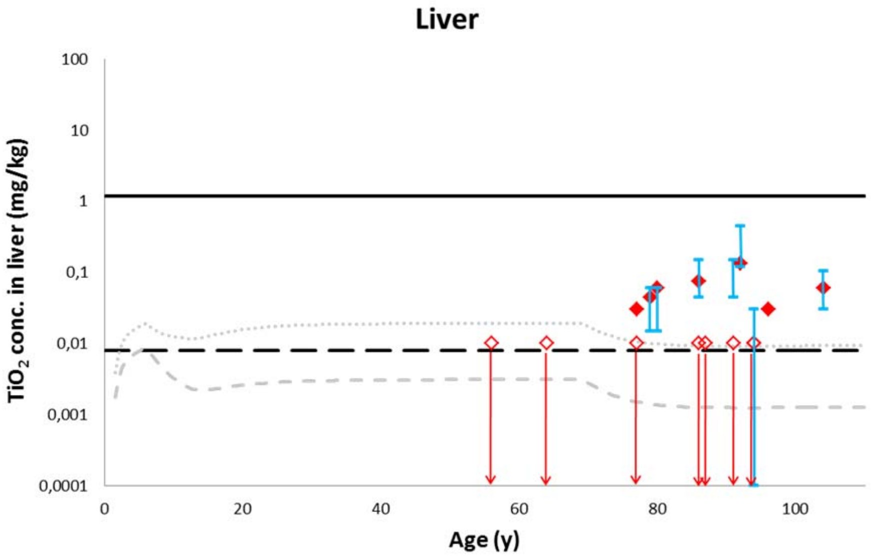

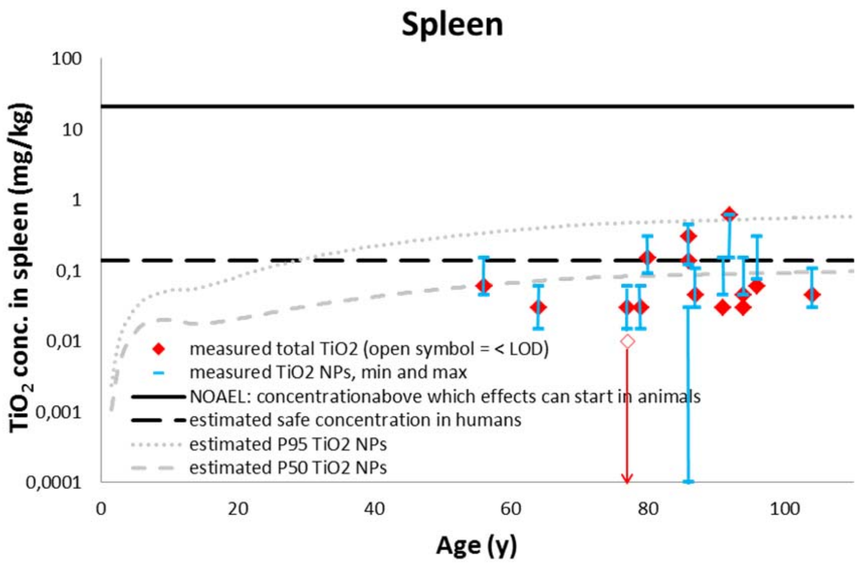

Figure S1. Measured and modelled total Ti (expressed as TiO_2 to enable comparison) and TiO_2 particle concentrations in human liver and spleen as a function of age, together with liver or spleen concentrations that are relevant for risk assessment. Red diamonds: measured total Ti concentration in human post-mortem liver or spleen (expressed as TiO_2), with open diamonds and arrows representing the possible levels in the samples where the Ti level was below the limit of detection (LOD). Blue lines: range between measured minimum and maximum particle TiO_2 concentrations (i.e. uncorrected and corrected for analytical recovery; expressed as a concentration on weight basis, mg/kg tissue, to enable comparison) in human post-mortem liver or spleen. Black solid line: internal concentration of 21 mg TiO_2/kg spleen that corresponds to no effects at the top dose tested (200 mg/kg bw/d), or an internal concentration of 1.2 mg TiO_2/kg liver that corresponds to an external dose of 10 mg/kg bw/day (point of departure; PoD) above which liver damage and edema in animals were observed[3]. Black dashed line: internal concentration of 0.14 mg TiO_2/kg (spleen) and 0.008 mg TiO_2/kg (liver), which is estimated to be safe for humans based on the PoD and considering various assessment factors. Grey lines: modelled concentration of TiO_2 nanoparticles in humans based on an estimated daily intake of the P95 (•••) or P50 (- - -) of the population and the toxicokinetic model developed by Heringa et al[1]. Intake for different age groups as well as growth was taken into account resulting in the changes in the course of the modelled TiO_2 liver and spleen concentrations at specific ages.

5. References

1 Heringa, M. B. *et al.* Risk assessment of titanium dioxide nanoparticles via oral exposure, including toxicokinetic considerations. *Nanotoxicology* **10**, 1515-1525, doi:10.1080/17435390.2016.1238113 (2016).

2 Bettini, S. *et al.* Food-grade TiO2 impairs intestinal and systemic immune homeostasis, initiates preneoplastic lesions and promotes aberrant crypt development in the rat colon. *Sci Rep* **7**, 40373, doi:10.1038/srep40373 (2017).

3 Wang, Y. *et al.* Susceptibility of young and adult rats to the oral toxicity of titanium dioxide nanoparticles. *Small* **9**, 1742-1752, doi:10.1002/smll.201201185 (2013).

4 Urrutia-Ortega, I. M. *et al.* Food-grade titanium dioxide exposure exacerbates tumor formation in colitis associated cancer model. *Food Chem Toxicol* **93**, 20-31, doi:10.1016/j.fct.2016.04.014 (2016).

5 Shukla, R. K. *et al.* TiO2 nanoparticles induce oxidative DNA damage and apoptosis in human liver cells. *Nanotoxicology* **7**, 48-60, doi:doi:10.3109/17435390.2011.629747 (2013).

6 van Leeuwen, C. J. & Vermeire, T. G. 686 (Springer Dordrecht (the Netherlands), 2007).

7 Rompelberg, C. *et al.* Oral intake of added titanium dioxide and its nanofraction from food products, food supplements and toothpaste by the Dutch population. *Nanotoxicology* **10**, 1404-1414, doi:10.1080/17435390.2016.1222457 (2016).

8 Powell, J. J., Faria, N., Thomas-McKay, E. & Pele, L. C. Origin and fate of dietary nanoparticles and microparticles in the gastrointestinal tract. *J Autoimmun* **34**, J226-233, doi:10.1016/j.jaut.2009.11.006 (2010).

9 Brun, E. *et al.* Titanium dioxide nanoparticle impact and translocation through ex vivo, in vivo and in vitro gut epithelia. *Particle and fibre toxicology* **11**, 13, doi:10.1186/1743-8977-11-13 (2014).

10 Kreyling, W. G. *et al.* Quantitative biokinetics of titanium dioxide nanoparticles after intravenous injection in rats: Part 1. *Nanotoxicology* **11**, 434-442, doi:10.1080/17435390.2017.1306892 (2017).

11 Kreyling, W. G. *et al.* Quantitative biokinetics of titanium dioxide nanoparticles after oral application in rats: Part 2. *Nanotoxicology* **11**, 443-453, doi:10.1080/17435390.2017.1306893 (2017).

12 Geraets, L. *et al.* Tissue distribution and elimination after oral and intravenous administration of different titanium dioxide nanoparticles in rats. *Part Fibre Toxicol* **11**, 30, doi:10.1186/1743-8977-11-30 (2014).

13 Bello, D. & Warheit, D. B. Biokinetics of engineered nano-TiO2 in rats administered by different exposure routes: implications for human health. *Nanotoxicology* **11**, 431-433, doi:10.1080/17435390.2017.1330436 (2017).
